# Noncrystallographic symmetry-constrained map obtained by direct density optimization

**DOI:** 10.1107/S2059798319017297

**Published:** 2020-01-31

**Authors:** Masato Yoshimura, Nai-Chi Chen, Hong-Hsiang Guan, Phimonphan Chuankhayan, Chien-Chih Lin, Atsushi Nakagawa, Chun-Jung Chen

**Affiliations:** a Life Science Group, Scientific Research Division, National Synchrotron Radiation Research Center, 101 Hsin-Ann Road, Hsinchu 30076, Taiwan; b Institute for Protein Research, Osaka University, 3-2 Yamadaoka, Suita, Osaka 565-0871, Japan; c Department of Biotechnology and Bioindustry Sciences, National Cheng Kung University, Tainan 701, Taiwan; d Department of Physics, National Tsing Hua University, Hsinchu 30043, Taiwan

**Keywords:** noncrystallographic symmetry, NCS-constrained map, direct density optimization, twinning, *REFMAC*5

## Abstract

Noncrystallographic symmetry (NCS) averaging following molecular-replacement phasing is generally the major technique used to solve a structure with several molecules in one asymmetric unit, such as a spherical icosahedral viral particle. As an alternative method to NCS averaging, a new approach to optimize or to refine the electron density directly under NCS constraints is proposed. This method has the same effect as the conventional NCS-averaging method but does not include the process of Fourier synthesis to generate the electron density from amplitudes and the corresponding phases. It has great merit for the solution of structures with limited data that are either twinned or incomplete at low resolution. This method was applied to the case of the *T* = 1 shell-domain subviral particle of *Penaeus vannamei* nodavirus with data affected by twinning using the *REFMAC*5 refinement software.

## Abstract 2

A noncrystallographic symmetry-constrained map obtained by direct density optimization is efficient and equivalent to a noncrystallographic symmetry-averaging map. Using the noncrystallographic symmetry-constrained map, the structure of a *T* = 1 *Penaeus vannamei* nodavirus shell-domain subviral particle was newly determined by including twinned data.

## 1. Introduction   

The molecular-averaging method in real space coupled with solvent flattening is powerful in phase determination or phase improvement in protein crystallography. In the structure determination of icosahedral viruses, noncrystallographic symmetry (NCS) averaging with phase extension is a common procedure for phase improvement after initial calculations based on molecular replacement (MR) using a density map from a cryo-electron microscope, a similar structural model or initial experimental phases from isomorphous replacement or anomalous dispersion (Arnold *et al.*, 1987 ▸). Under the special conditions that the envelope or icosahedral matrices are given with sufficient precision and the degrees of freedom of the density are sufficiently small, *i.e.* a lower crystallographic free fraction, the map can be built *ab initio* (Yoshimura *et al.*, 2016 ▸). Since early in the 1970s, molecular averaging has been performed with iterative calculations of Fourier transformation (FT) and inverse FT between real and inverse space (Buehner *et al.*, 1974 ▸; Bricogne, 1976 ▸). Many applications and results using iterative molecular-averaging methods have been reviewed by Kleywegt & Read (1997 ▸).

As an alternative method, we have conceived a method to optimize or refine the density values directly under NCS constraints to reproduce the observables of the amplitude *F*_obs_. We know empirically that such a direct density-optimization (DDO) method has the weakness of a small convergence radius and difficulty in finding the initial conditions to achieve the correct solutions, but once the initial conditions have been obtained, it has a good potential to attain the most reasonable solution. In contrast to the conventional averaging method, the DDO method contains no Fourier synthesis, in which the electron density is calculated from the amplitudes (usually the observables *F*_obs_) and the corresponding phases. Consequently, the electron-density maps do not suffer from incomplete observables, which are typically caused by the experimental setup, such as a cut by the beamstop shadow. Furthermore, in cases of twinned data there is significant merit in avoiding the process of Fourier synthesis, in which detwinning (or deconvoluting) the amplitudes is generally difficult.

In this paper, we report the principles and the application of the DDO method to construct an NCS-constrained density map of the *T* = 1 shell-domain (S-domain; Sd) subviral particle (SVP) of *Penaeus vannamei* nodavirus (*Pv*NV) in order to solve its structure. The biological details and structural results of *Pv*NV have recently been reported (Chen *et al.*, 2019 ▸). We obtained two crystal forms, in space groups *P*2_1_2_1_2_1_ and *P*2_1_3, for the *T* = 1 *Pv*NV S-domain SVP, where the data from the *P*2_1_3 crystal were merohedrally twinned. The coordinates of the *T* = 1 S-domain SVP of Grouper nervous necrosis virus (GNNV; PDB entry 4rft; Chen *et al.*, 2015 ▸) served as an initial model; the NCS-constrained electron-density maps of the two crystal forms were obtained using the DDO method. Using the NCS-constrained maps, a new structure of the *T* = 1 *Pv*NV S-domain SVP was determined. Even though one data set was twinned, the electron-density map was deduced. In the following, we discuss the DDO method, the conditions for acquiring valid electron density and the convergence radius, and compare the DDO method with other common methods.

## 2. Concept and methods   

### 2.1. Concept   

**Figure 1:**
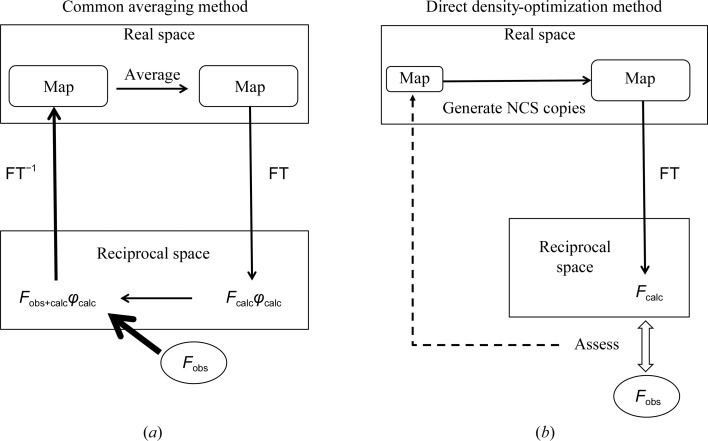
Schematic diagrams for the two types of calculations: (*a*) conventional iterative methods for NCS averaging and (*b*) the direct density-optimization (DDO) method to generate the NCS-constrained map.

**Figure 2:**
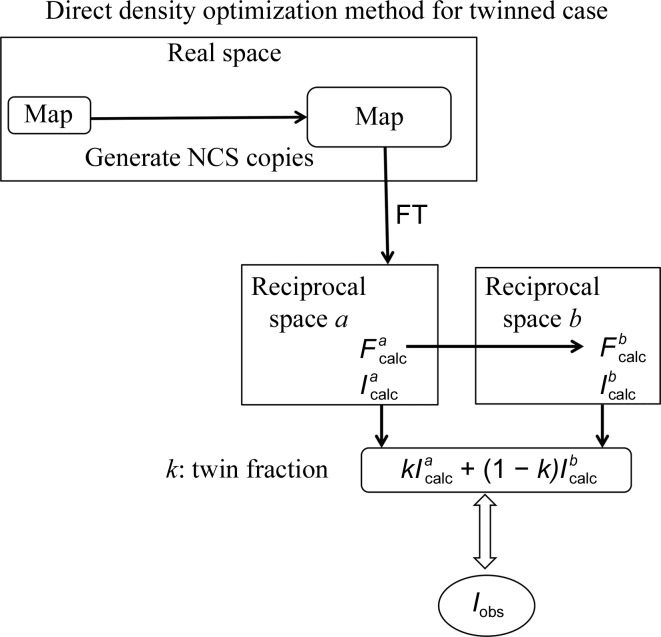
Schematic diagram for the direct density-optimization (DDO) method in the case of twinning.

Molecular averaging in real space is generally performed with an iterative calculation between real and inverse space linked by Fourier transformation (FT) and inverse FT (FT^−1^). A schematic diagram of this common method is shown in Fig. 1 ▸(*a*). Here, we propose a new method according to which the density of the minimal region for a target molecule is first postulated and copies of NCS-equivalent molecule densities are then generated. The calculated amplitudes (*F*_calc_) produced by FT are eventually compared with the observed amplitudes (*F*_obs_), as shown in Fig. 1 ▸(*b*). To maximize the agreement between the calculated *F*_calc_ and the data *F*_obs_, the density of the molecule is hence treated as a parameter and optimized. This method is free from the Fourier synthesis (FT^−1^) process that uses the data amplitude *F*_obs_ (Fig. 1 ▸). It has the merit of treating twinned data and solving the structure without difficulty (Fig. 2 ▸). This concept can be recognized as a ‘refinement’ technique that is directly applied to the electron density. To obtain valid electron density, the correct region of the target molecule, the correct NCS matrices and a sufficiently small crystallographic free fraction (ff; Yoshimura *et al.*, 2016 ▸), which denotes the ratio of the unconstrained density region to the unit-cell volume, are essential. These conditions are described in Section 4. Table 1 ▸ lists notations for and definitions of the terms and abbreviations used in this work.

### 2.2. Method for DDO calculations   

As there was no suitable DDO software for us to directly optimize or to refine the electron-density values in this work, we utilized the popular refinement program *REFMAC*5 (Murshudov *et al.*, 2011 ▸) to perform and test the DDO method in an indirect way. *REFMAC*5 refines the coordinate parameters and temperature factors in seeking the maximum likelihood to reproduce the amplitude observables. The solution from the MR method was used for the initial density. The solution using the GNNV SVP retained the icosahedral relation. An icosahedral unit of the molecule was selected; the other molecules were expressed with each NCS matrix, and the NCS matrices were used as the NCS-constraint parameters for the refinement. In the case of our icosahedral virus structures, strict icosahedral matrices were constantly applied as the NCS matrices. Using *REFMAC*5, the coordinates and *B* factors of the initial model were refined under unrestrained refinement, while imposing NCS constraints using the ncsconstraint option. Using unrestrained refinement that is free of any restriction by local geometry restraints, such as bond lengths and bond angles, the common case is to move atom positions that are meaninglessly dispersed because of the overfitting condition. In our case of unrestrained refinement while imposing many NCS constraints, atomic coordinates were also dispersed such that the atoms were no longer present at the actual atomic positions. Nevertheless, when the *R* factor was decreased sufficiently even under many NCS constraints, we obtained interpretable electron density that was generated with the dispersed coordinates and *B* factors of atoms. The obtained density map, which was generated with calculated amplitudes *F*_calc_ and phases, was determined as an ‘NCS-constrained map’. If necessary, the parameters of the NCS matrices can be refined by monitoring the decreasing *R* factors.

### 2.3. Method for purification, crystallization and data collection   

Orthorhombic (*P*2_1_2_1_2_1_) and cubic (*P*2_1_3) crystals of ΔN-ARM *T* = 1 *Pv*NV S-domain SVP, hereafter denoted *T* = 1 *Pv*NV-Sd, were obtained by the hanging-drop vapour-diffusion method. Drops containing the *T* = 1 S-domain subparticle (30 mg ml^−1^ in 50 m*M* HEPES pH 7.4, 300 m*M* NaCl, 5 m*M* CaCl_2_) were allowed to equilibrate at 18°C against reservoirs consisting of (i) 0.1 *M* Tris pH 8.0, 20%(*w*/*v*) poly(acrylic acid sodium salt) 5100 or (ii) 0.1 *M* Tris pH 7.8, 0.2 *M* l-arginine, 8%(*w*/*v*) poly-γ-glutamic acid. All crystals grew after two weeks. X-ray diffraction data from these two crystal forms, cryoprotected with 20% glycerol, were collected at 100 or 110 K on beamline BL44XU at SPring-8, Harima, Japan and beamline TPS 05A at NSRRC, Hsinchu, Taiwan. The data were indexed and processed using *HKL*-2000 (Otwinowski & Minor, 1997 ▸). The data resolutions for the *P*2_1_2_1_2_1_ and *P*2_1_3 crystals was 3.38 and 3.30 Å, respectively, in the initial phasing stage. The data with the best resolution of 3.12 Å for the *P*2_1_2_1_2_1_ crystal form were obtained later during the final structure-refinement stage for the structure report (Chen *et al.*, 2019 ▸). The data statistics are shown in Table 2 ▸.

## 3. Results   

### 3.1. Single-crystal data in space group *P*2_1_2_1_2_1_   

Initial phasing of the data in space group *P*2_1_2_1_2_1_ was performed by the MR method using the structure of *T* = 1 GNNV SVP (PDB entry 4rft) as the initial model (Chen *et al.*, 2015 ▸) in *Phaser* (McCoy *et al.*, 2007 ▸). One *T* = 1 SVP, which consists of 60 copies of the S-domain, was estimated to be included in the NCS unit. *Phaser* found an MR solution that showed a log-likelihood gain (LLG) score of 570 (the LLG before refinement was 35) on inputting an identity parameter (IDENT) of 0.5. The initial weighted (2*F*_o_ − *F*_c_) map with the MR phases was completely uninterpretable (Supplementary Fig. S1). From the initial MR solution of the SVP of GNNV, we obtained the initial 60-fold NCS matrices that maintained the strict icosahedral form. Using the *REFMAC*5 refinement software (Murshudov *et al.*, 2011 ▸), the atomic coordinates and temperature factors of a single S-domain (a minimal icosahedral unit) were optimized by ‘unrestrained refinement’ while imposing 60-fold ‘constrained’ NCS matrices. The number of non-H atoms in the GNNV S-domain was 1248. After the unrestrained refinement, the atomic coordinates of the GNNV S-domain completely dispersed: almost all of the atomic coordinates did not reflect the new correct atom positions. Despite the dispersed atomic coordinates, *REFMAC*5 showed a reasonable *R* factor; in this case it was less than 25%. The *R* factor typically shows the agreement of the calculated amplitudes from the atomic model with the diffraction data. In this work, we name the *R* factor the ‘*R*_d_ factor’ to represent the agreement of the calculated amplitudes from the electron density, instead of the atomic model, with the observed data.

**Figure 3:**
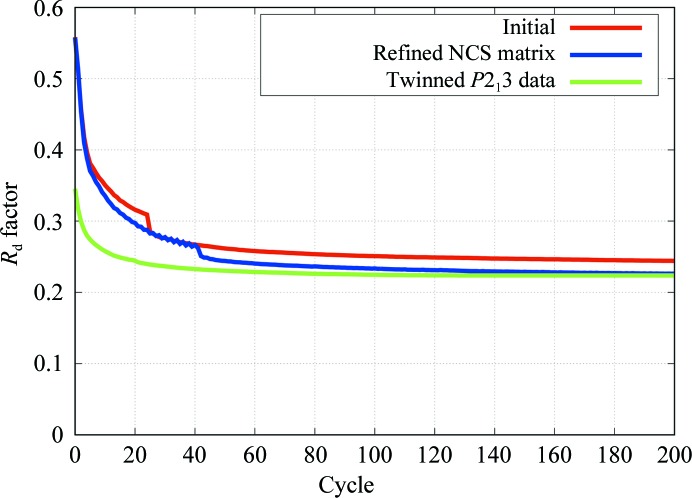
*R*_d_ factors plotted against refinement cycles. The red line shows the results with the initial NCS matrices for the single-crystal *P*2_1_2_1_2_1_ data. The blue line shows the results with the refined icosahedral NCS matrices. The green line shows the results with the twinned *P*2_1_3 data. All results are for data at the phasing stage.

**Figure 4:**
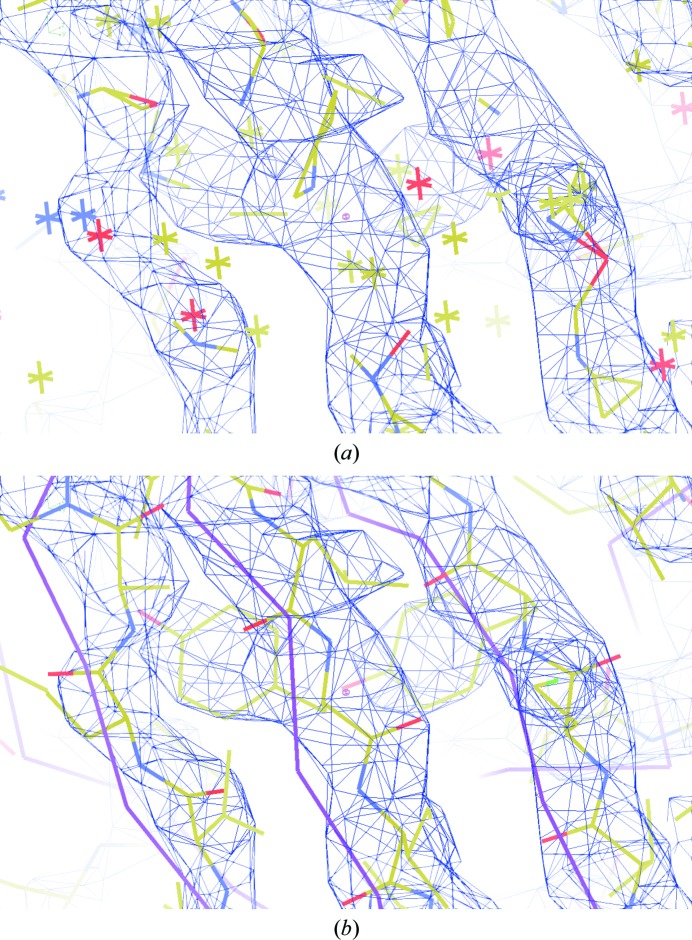
(*a*) The NCS-constrained electron-density map with dummy atoms that are fully dispersed from the actual atomic positions (yellow sticks) contains the correct electron-density information. (*b*) The NCS-constrained map with the main-chain trace of the initial model of GNNV (purple) and the final structural model of *Pv*NV-Sd (yellow sticks) fitted to the map as a guide for the eye. Using the NCS-constrained map, the structure model was built. The bias from the initial MR model can be removed. The contour level of the map is 2.3σ.

The number of unrestrained refinement cycles was extended as long as the *R*_d_ factor decreased and converged to a minimum value. The number of cycles was usually less than 200. On monitoring the *R*_d_ factors, initial parameters, such as NCS matrices, were refined with perturbation trials and as few as ten refinement cycles. In Fig. 3 ▸, these *R*_d_ factors with initial NCS matrices and with refined NCS matrices are shown against the cycle number of the *REFMAC*5 refinement. When refinement of the NCS matrices was performed, the 60-fold NCS matrices were maintained to retain a strict icosahedral relation to each other; *i.e.* only six parameters for rotations and positions of the centre of the icosahedron to generate NCS matrices were refined. After the NCS matrices had been refined, we obtained an NCS-constrained map with an *R*_d_ factor of 23%, which was generated from ‘calculated amplitudes *F*_calc_’ and ‘calculated phases’. Using the data with the highest resolution of 3.12 Å, the NCS matrix of the icosahedron was refined again and the final *R*_d_ factor reached 21%. As the refined atomic coordinates did not reflect the actual atomic positions of the structure, we call these refined atoms ‘dummy atoms’. The dispersed dummy atoms that served the original MR model are shown in Fig. 4 ▸(*a*) with the obtained NCS-constrained map. With the NCS-constrained map, the new structure of the *T* = 1 *Pv*NV-Sd particle in the icosahedral unit could be manually built with *Coot* (Emsley *et al.*, 2010 ▸). The built model in the icosahedral unit underwent restrained refinement under the final NCS constraints that maintained strict icosahedral relations (PDB entry 5yl1). The *R*_work_ and *R*_free_ of the final model were 21.9% and 22.1%, respectively (Chen *et al.*, 2019 ▸; Table 2 ▸). The main chains of the initial MR model (*T* = 1 GNNV SVP; PDB entry 4rft), the final refined structure model (*T* = 1 *Pv*NV-Sd) and the NCS-constrained map are shown in Fig. 4 ▸(*b*).

### 3.2. Twinned crystal data in space group *P*2_1_3   

According to the statistics of the amplitudes and the icosahedral-related peaks from the self-rotation function, the data in space group *P*2_1_3 were expected to be merohedrally twinned. All estimators of twinning obtained using *TRUNCATE* in the *CCP*4 software package (Winn *et al.*, 2011 ▸) showed that the data were completely twinned; for example, an *L* statistic of 0.339 (Padilla & Yeates, 2003 ▸). The self-rotation function showed two directions of icosahedral symmetry, with each orientation related by a 90° rotation. From the crystal symmetry and packing analysis, we located the centres of four presumed particles at (1/4, 1/4, 1/4), (3/4, 3/4, 1/4), (1/4, 3/4, 3/4) and (3/4, 1/4, 3/4). One-third of the *T* = 1 SVP, which contains 20 copies of *Pv*NV-Sd, was present in the NCS unit.

**Figure 5:**
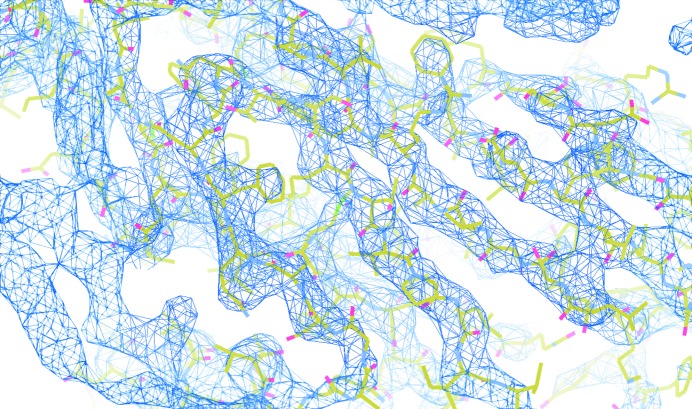
The NCS-constrained map from the merohedrally twinned data. The map is interpretable and clear despite the twinning. The contour level of the map is 2.0σ. The final refined structure model of *Pv*NV-Sd fitted to the map is shown in yellow as a guide for the eye.

Using the refined dummy atoms, which contained the information from the electron density of *Pv*NV-Sd but in which the atomic positions seemed to be dispersed, the initial phases from MR for the twinned data from the *P*2_1_3 crystal were obtained with a high LLG score (IDENT = 0.8) that was as large as 10 000 on refining the rotations and translations of 20 initial molecules with refinement and phasing by *Phaser*. As a trial, using the *T* = 1 GNNV SVP as the initial structural model, *Phaser* picked very low LLG scores of ∼20 (IDENT = 0.8). One dummy-atom molecule of *Pv*NV-Sd from the MR solution was selected; its NCS matrix was determined as a unit matrix. Using the other 19 NCS-related molecules of the solution, the 19 NCS matrices were calculated. The DDO method only has a convolution process of amplitude calculation for twinned observables. *REFMAC*5 has a refinement function for the twinning fraction (see Section 2.3 of Murshudov *et al.*, 2011 ▸); this showed that the twinning operator was (*k*, *h*, −*l*) against (*h*, *k*, *l*). Using *REFMAC*5, the dummy atoms were subjected to unrestrained refinement with 20 NCS constraints and with twinning-fraction refinement. The *R*_d_ factor of the unrestrained refinement reached 22.4% (Fig. 3 ▸). The twinning fractions were 0.69 and 0.31 in the final refinement cycle. The NCS-constrained maps for twinned data with the final refined model of *Pv*NV-Sd are shown in Fig. 5 ▸.

## 4. Discussion   

### 4.1. Direct density optimization and *REFMAC*5   

Since the early years, NCS has been known to be powerful in phase improvement (Main & Rossmann, 1966 ▸; Bricogne, 1976 ▸; Arnold & Rossmann, 1986 ▸; Rossmann, 1990 ▸), and has assisted in many cases of phase improvement using several programs, such as *DM* (Cowtan & Main, 1996 ▸) in the *CCP*4 suite and *RESOLVE* (Terwilliger, 2002 ▸) in *Phenix* (Liebschner *et al.*, 2019 ▸). Recent attention has been paid to real-space refinement because of the requirements of cryo-EM structure refinement (Afonine *et al.*, 2018 ▸). In almost all cases, the imposition of NCS has been performed more or less using the iterative or averaged method shown in Fig. 1 ▸(*a*). Fourier synthesis calculations (or inverse Fourier transformations; FT^−1^) are required to process the map, whereas the DDO method has no Fourier synthesis calculation (Fig. 1 ▸ *b*). A map generated using Fourier synthesis calculations could be affected by defects in the data. The strength of the DDO method is its robustness against defects in the data *F*_obs_, such as statistical errors, systematic noise and incompleteness. Especially in cases of twinning, the decomposition of amplitudes is a difficult task in the Fourier synthesis calculations in the common iterative-averaging method. The DDO method has a great strength in avoiding this difficulty in the decomposition of observables.

To execute the DDO method, we used the refinement function of *REFMAC*5, which refines the atomic coordinates and their temperature factors. Our initial intent was to seek to undertake refinement of the temperature factors or occupancies of fixed coordinate atoms, which served to refine densities on the map grid positions. We found, unexpectedly, that flexible atomic coordination was not a problem in obtaining correct densities under the conditions described in the next section. We previously reported that the envelope of the region is important for NCS averaging (Yoshimura *et al.*, 2016 ▸). The condition that we can refine the coordinates of density grid points means that we can refine the envelope itself with increased flexibility.

### 4.2. Conditions for obtaining a correct map   

In our previous work (Yoshimura *et al.*, 2016 ▸), we introduced the crystallographic free fraction (ff), which gives the ratio of the unconstrained density region to the unit cell. When ff is sufficiently small, which is the condition of over-determination, the density of the unconstrained region is determined uniquely. We showed an empirical ff value of <0.1 to be the criterion for a feasible *ab initio* condition using the iteration-averaging method. In this work, we show the same situation for the feasible direct determination of the density. The values of ff are <1/60 for the *P*2_1_2_1_2_1_ data and <1/20 for the twinned *P*2_1_3 data; the inequality arises from considering the constraint on the solvent region. Both ff values are sufficiently small. Even the number of dummy atoms (1248), which was the original number of non-H atoms in the GNNV S-domains, has an additional degree of freedom of the *x*, *y* and *z* coordinates in the *REFMAC*5 refinement. The condition of over-determination remained satisfied when the number of variables (4996; 1248 × 4) was 1/21 times the number of unique observables (206 891) in the *P*2_1_2_1_2_1_ data. Two further conditions, the correct NCS matrices and NCS minimal regions and the acquisition of a global minimum unique solution, are also required to derive a correct map. We summarize these three points as

(i) correct NCS matrix and region,
(ii) sufficiently small ff,
(iii) acquisition of a global minimum solution.

When the value of ff is not sufficiently small but satisfies ff < 0.5, even on obtaining a global minimum solution or a unique solution (Millane & Arnal, 2015 ▸) care should be taken in obtaining the density map in order to avoid it being ill-posed through errors in the data.

To examine whether a global minimum solution can be obtained, we refer to the metric of agreement between the calculated amplitudes and observables as the *R*_d_ factor; we can then evaluate the solution by inspecting whether or not the electron-density map is interpretable. A starting point or an initial condition from which the global minimum can be attained is referred to as ‘in the convergence radius’. When the parameter optimization falls into a local minimum or a shortage of the degree of freedom of the parameters occurs, the third condition of the acquisition of a global minimum solution is not satisfied.

### 4.3. Convergence radius of the DDO method   

In contrast to the iteration-averaging methods, the convergence radius of direct density refinement is empirically expected to be small. The small values of ff and the precise icosahedral NCS matrices guided the derivation of the correct density. Fortunately, we attained a global minimum solution for the *P*2_1_2_1_2_1_ single-crystal data beginning from the initial condition of the GNNV S-domain particles. So far, for the twinned *P*2_1_3 data we only obtained a correct solution when using *Pv*NV dummy atoms as the starting point, for which the initial *R*_d_ factor was 35% (Fig. 3 ▸). Our few trials using GNNV S-domain particles as a starting point did not succeed for the twinned data, which might reflect a small convergence radius for the DDO method. The other reason is that the twinning-fraction parameter led to a moderate increase in ff (<1/20) because of the additional degrees of freedom of the variables. Phasing of the twinned crystal is a major problem to be overcome (Ginn & Stuart, 2016 ▸; Sabin & Plevka, 2016 ▸); we need to further investigate how to increase the convergence radius to obtain a global minimum solution.

### 4.4. Comparison with the common iterative-averaging method   

**Figure 6:**
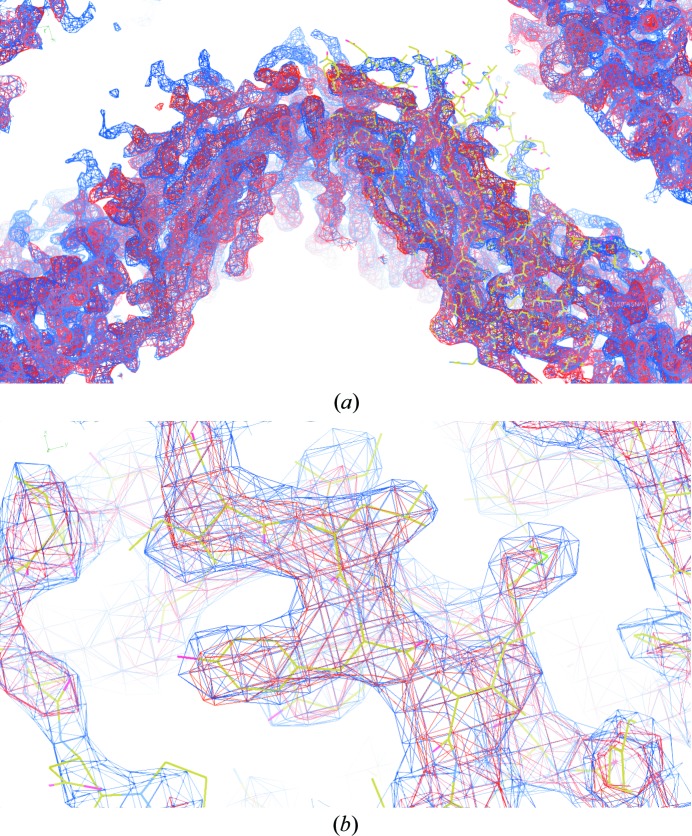
A comparison of the NCS-constrained map (blue mesh) with the *DM* map (red mesh) at the same contour level (1.5σ). Both maps were calculated using the 3.37 Å resolution data at the phasing stage and initial NCS matrices which were obtained from the MR solution. (*a*) shows a view of the fivefold axis of the viral particles. The outside of the particle in the *DM* map (red) disappears owing to the initial NCS masks. (*b*) Both maps have the same quality; the NCS-constrained map is a little denser. The yellow stick model is the final model fitted to the map as a guide for the eye.

In Fig. 6 ▸, we compare the conventional NCS-averaging map from the *DM* software with the map from the DDO method. There is no critical difference in quality between the two maps. However, in Fig. 6 ▸(*a*) there are missing densities for the *DM* map on the outside surface of the *T* = 1 *Pv*NV-Sd particle which might not be covered by the NCS mask. For *DM*, it is difficult to generate the NCS (solvent or averaging) mask at the boundary regions between neighbouring particles. The DDO method has the same function of solvent flattening as *DM* in placing no dummy atoms in the solvent region. Such a vacant space for dummy atoms is fixed to a density value of zero. The DDO method has flexibility in the boundary-region determination because of the atom mobilities. *DM* includes an additional function of histogram matching, but the DDO method does not apply such a process. Furthermore, *DM* can execute averaging in a phase-extension manner to guide towards a global minimum solution, whereas no such equivalent function can be applied in the DDO method. The incorporation of phase extension into the DDO method will be our next work, in which the convergence radius will be expected to increase.

### 4.5. Comparison with *REFMAC*5 restrained refinement   

**Figure 7:**
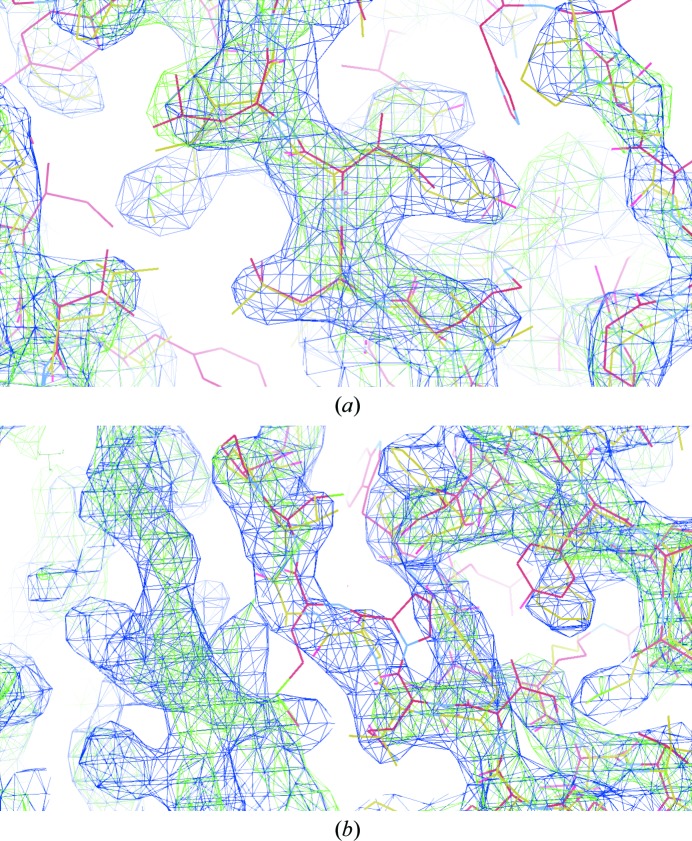
Comparisons of the DDO map (blue mesh) with the map resulting from *REFMAC*5 restrained refinement (green mesh). The *REFMAC*5 map (green mesh) is the weighted (2*F*_o_ − *F*_c_) map. The contour levels of both maps are 2.0σ. Both maps were calculated using the 3.37 Å resolution data at the phasing stage and the initial NCS matrices which were obtained from the MR solution. Yellow carbon bonds are the final model fitted to the NCS-constrained map. Brown carbon bonds are the result of *REFMAC*5 restrained refinement. C^α^ displacements between the final fitted model and the MR solution are 2.7 Å on average. Most of the refined main chains (brown) jumped to fit to the new position, as shown in (*a*). However, some parts of the refined main chains failed to fit the new map, as shown at the centre of (*b*). Even though both maps were calculated using the same initial structure, the NCS-constrained map from DDO is free from bias from the initial structure. The yellow stick model is the final model fitted to the map as a guide for the eye.

In Fig. 7 ▸, we compare the DDO map with the resulting map from *REFMAC*5 refinement with the common use of coordinates restrained by the 60-fold NCS constraint. The restrained refinement was performed with sufficient cycles (200). Although there was a structural difference of 2.7 Å on average between the main chains of the initial MR model and the final built model, most fractions of the main chains of the MR model were successfully shifted and adjusted to the new correct positions after refinement. The *R* factor had a smallest value of 0.465 when the ridge distance σ was 0.02 (from the range 0.01–0.30) for the ‘jelly-body’ restraint function (see Section 2.6.5 of Murshudov *et al.*, 2011 ▸). Most of the main chain seemed to be traceable with the weighted (2*F*_o_ − *F*_c_) map by *REFMAC*5. Most parts of the main chains were successfully shifted to new main-chain positions, as shown in Fig. 7 ▸(*a*), but some parts failed to be shifted, as shown in Fig. 7 ▸(*b*). The NCS-constrained map from DDO can be seen to easily provide a completely new phase, removing the bias from the initial model phases by completely loosening the restraints on coordinates. In Table 3 ▸, the mean phase error and *R*_d_ factor for each method are summarized, showing that the removal of bias in the DDO method and *DM* is of comparable quality.

## 5. Conclusions   

The use of NCS is powerful for phasing or phase improvement. NCS has been used as an averaging method in real space. We propose an NCS-constrained map with a new method of direct density optimization (DDO). Whereas the NCS-constrained map is equivalent to an NCS-averaging map, the DDO method requires no Fourier synthesis that calculates a map from *F*_obs_ and its phases. To perform DDO, we used unrestrained refinement in *REFMAC*5 while imposing NCS constraints, and applied this method to a new structure of *T* = 1 *Pv*NV-Sd. With the condition that the crystallo­graphic ff is sufficiently small, an interpretable map was obtained and the structure of *T* = 1 *Pv*NV-Sd was subsequently solved. We further demonstrate the application of the DDO method to other *T* = 1 *Pv*NV-Sd data sets that were twinned, in which the DDO method has no difficulty in generating the NCS-constrained map. A comparison of the NCS-constrained map with DDO and the map from *DM*, which uses the averaging method, shows no critical difference except that the DDO map produces more complete density at the molecular boundary. Compared with the result of *REFMAC*5 restrained refinement, the NCS-constrained map with DDO can easily remove the bias from the initial model with the same effort. By making the convergence radius large in future developments, the method will be more powerful for the solution of structures with a large number of NCS including twinned data.

## Supplementary Material

Supplementary Figure S1. DOI: 10.1107/S2059798319017297/ji5009sup1.pdf

## Figures and Tables

**Table:** Abbreviations.

DDO	Direct density optimization	
*Pv*NV	*Penaeus vannamei* nodavirus	New virus to be solved in this work
GNNV	Grouper nervous necrosis virus	Similar virus model used in MR phasing
SVP	Subviral particle	
*Pv*NV-Sd	*Pv*NV shell-domain (S-domain)	New structure to be solved in this work
ff	Crystallographic free fraction†	

**Table:** Special terms.

ff	Ratio of unconstrained region volume to the total unit cell†
*R* factor	*R* factor with *F*_calc_ from model
*R*_d_ factor	*R* factor with *F*_calc_ from electron-density map. In this work, it is calculated using a dummy-atoms model. The derivation is the same as that of the *R* factor.
Dummy atoms	Pseudo-atoms which do not reflect actual atoms or merely show the coordinates for positions that have some electron-density values and contributions.

† Yoshimura *et al.* (2016 ▸).

**Table 2:** Data collection and processing Values in parentheses are for the outer shell.

	*T* = 1 ΔN-ARM *Pv*NV-Sd SVP (PDB entry 5yl1)		
	Phasing	Refinement	*T* = 1 *Pv*NV-Sd SVP
Data collection			
Diffraction source	BL44XU, SPring-8	TPS 05A, NSRRC	BL44XU, SPring-8
Wavelength (Å)	0.9792	1.0000	0.9000
Temperature (K)	100	110	100
Detector	MX300HE	MX300HS	MX300HE
Space group	*P*2_1_2_1_2_1_	*P*2_1_2_1_2_1_	*P*2_1_3
*a*, *b*, *c* (Å)	191.54, 196.86, 437.17	196.98, 200.32, 419.29	244.87
Resolution range (Å)	42.9–3.38 (3.56–3.38)	30–3.12 (3.23–3.12)	30–3.30 (3.42–3.30)
No. of unique reflections	206891	274292	69447
Completeness (%)	89.4 (6.0)	99.7 (99.8)	94.5 (69.3)
Multiplicity	6.1 (1.5)	5.0 (5.0)	5.6 (5.0)
〈*I*/σ(*I*)〉	6.0 (1.4)†	7.1 (1.7)†	4.8 (1.9)
Refinement			
*R*_work_/*R*_free_ (%)		21.9/22.1	
No. of non-H atoms			
Protein		1454	
Ions		2 [Ca^2+^]	
R.m.s. deviations			
Bonds (Å)		0.011	
Angles (°)		1.696	
Average *B* factors (Å^2^)			
Protein		64.6	
Ion		73.4	

† In the refinement, we forced 60-fold strict icosahedral constraints. This has the same effect as averaging.

**Table 3:** Comparisons with common methods in terms of mean phase errors and *R* factors The data at the phasing stage were used in these comparisons. In the mean phase-error calculations, the refined final structure model (PDB entry 5yli) with the data at the phasing stage was used as the best phases. The structure model 5yli was refined with the data to an *R* factor of 0.283 (*R*_free_ = 0.261). All *R* and *R*_d_ factor values are taken from the results of *REFMAC*5. Mean phase errors were calculated using *CPHASEMATCH* in *CCP*4 (Winn *et al.*, 2011 ▸).

Methods	Mean phase error (°)	*R* or *R*_d_ factor (free *R* or *R*_d_ factor)
Molecular replacement	76.0	0.559 (0.561)

	
NCS-constrained map by DDO	30.6	0.244 (0.246)
*REFMAC*5 restrained	52.6	0.465 (0.470)
*DM*	34.1	— (—)
