# Supplementary material for: Noncrystallographic symmetry-constrained map obtained by direct density optimization
Source: Acta Crystallogr D Struct Biol. 2020 Jan 31;76(Pt 2):147–54. doi: 10.1107/S2059798319017297 (PMC7008515; doi:10.1107/S2059798319017297)
Supplement: Supplementary file 1 [file d-76-00147-sup1.pdf]

STRUCTURAL
BIOLOGY

Volume 76 (2020)

Supporting information for article:

Noncrystallographic symmetry-constrained map obtained by direct density optimization

Masato Yoshimura, Nai-Chi Chen, Hong-Hsiang Guan, Phimonphan Chuankhayan, Chien-Chih Lin, Atsushi Nakagawa and Chun-Jung Chen

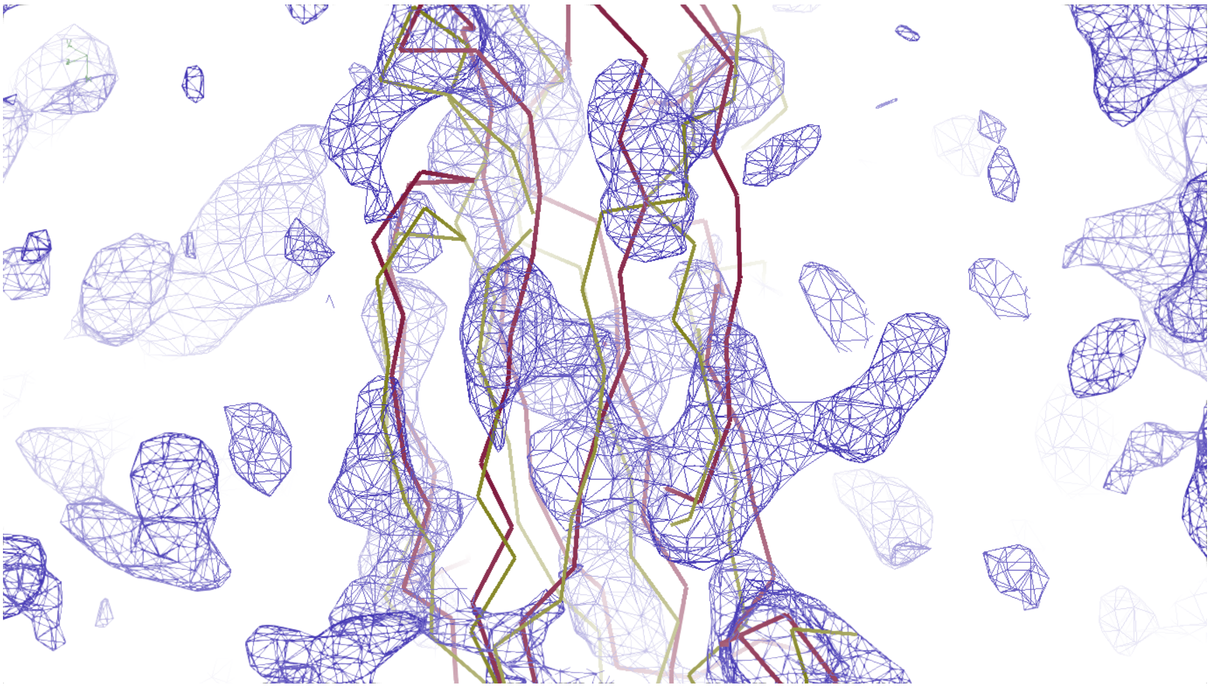

Figure S1 The initial map before NCS-constrained, *DM* or *REFMAC5* restraint calculations was applied. The map is weighted ($2F_o$-F_c) map of MR solution (*R*-factor = 55.9%). The contour level of map is 1.5 σ. The map is completely uninterpretable. Brown coloured line shows main-chains of the MR model. Yellow coloured one shows that of the final built model.
